# D11 and small angle neutron scattering: a paradigm of ILL

**DOI:** 10.1140/epje/s10189-023-00362-y

**Published:** 2023-11-13

**Authors:** John White

**Affiliations:** Research School of Chemistry, Australian National University, Canberra, 2603 Australia

## Abstract

This paper describes some of the early events in the 50 year success of D11. That, and the Institut Laue Langevin are linked by an attitude that embraces both instrument renewal and new areas of science. The neutron sources proved to be reliable and serviceable, the neutron guides and guide hall have been a great benefit, and the user concept has led to a strengthening international community of diverse and good science and to new instruments clamouring for funding in a growing facility. Designs were perfected and the second wind (Deuxième Souffle) funded the outflow from which a second strong phase has evolved.

**Graphical Abstract:**

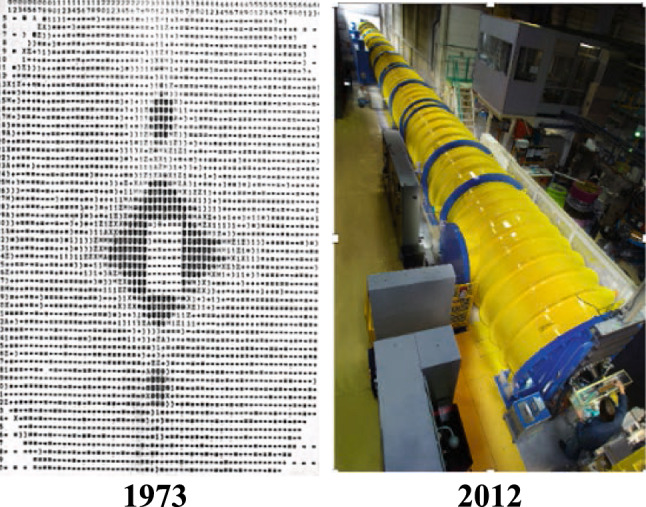

## Introduction

The Small Angle Scattering Instrument, D11, at the Institut Laue-Langevin (ILL), Grenoble, was a world leader at its inception in 1972, and has remained so. Constant technological renewal and scientific stimulus have ensured this. Publication rates, the diaspora of former D11 scientists and the D11 imitations elsewhere are characteristics of the whole Institut. This is my meaning of “a paradigm of the ILL” The whole Institut was established munificently by France and Germany at the highest level—a non-political symbol of the present and future unity of two countries. That munificence then, has engendered a great flowering of science and international collaboration.

Professor H Maier-Leibnitz’s imprint on the new Institut from 1967 endures. At the IAEA Symposium in Grenoble 6–10 March 1972 [1] he gave us a clue to the direction that neutron instrumentation was to take under his Direction: *…”an effort was made to develop and construct instrumentation that would allow adequate use of this costly neutron source. A compromise has been made between the wishes of the innovators and of the neutron experts who are mostly conservative. It has been an interesting time…”.*

Bernard Jacrot, his associate Director at the time, on page 64 of his book “Neutrons for Science” (p.64) [2], relates that Maier-Leibnitz’s aim was “an absence of a rigid hierarchy and, youthfulness of most of the “Actors,” which allowed an atmosphere to be created which was at the same time studious and relaxed”. “Very intense work by “Nice and clever people” was compensated by celebrations which were more or less improvised”. “Skiing between 12.00 and 14.00 occurred”.

D11 was not conservative and its use has been scientifically revolutionary. At the onset, the 80 m length, the movable “state of the art” LETI detector and later, the data processing at the instrument with mini-computers, allowed in situ “experiments” rather than measurements. The big tube and moveable detector have been widely copied as has the importance of skilled scientific and technical support for users [3].

**Fig. 1:**
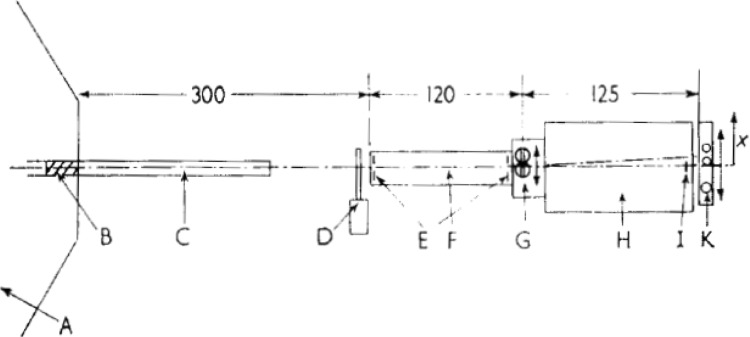
The Julich small angle scattering instrument with beryllium filtered neutrons, (dimensions of the collimators and flight tubes in metres)

The beginnings of D11 can be traced to the FRJ-2 (DIDO reactor at Julich) where many Munich reactor, Maier-Leibnitz students gravitated. The 1966 paper [4] by J. Christ, W.Schilling, W. Schmatz, and T. Springer, foreshadowed the D11 instrument. The diagram of that instrument is in Fig. 1.

On this instrument Schneider et al. [5] made the first SANS experiment finding the radii of gyration of deoxy- and oxy haemoglobulin (as 24.3 ± 1.6 Å and 23.8 ± 1.6 Å), carefully noting the effects of wave length spread on resolution. Apart from much science of the defect structure in metals and alloys this was a pioneering sally into biological structure with neutrons.

**Fig. 2:**
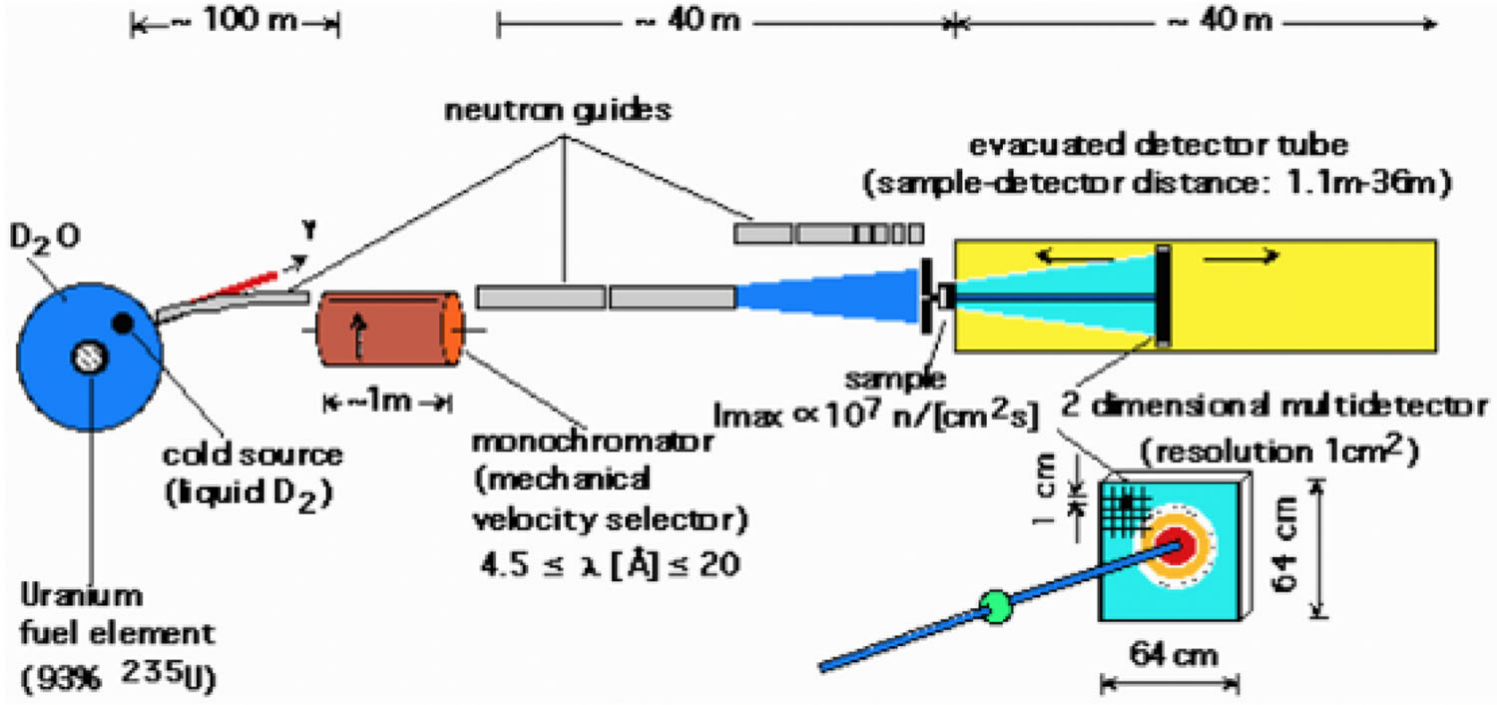
The D11 Instrument as Installed at the Institut Laue-Langevin

Figure 2 shows the final concept as reported by Ibel et al. [6]. Its availability caused a burst of new science from a broad scientific and technological base. This has continued for 50 years. Some users then come to mind: In Materials Science, G. Kostorz, W. Mitchell, in Polymers the French groups, H. Benoît, B. Farnoux, J. S. Higgins, G. Allen, G. Jannink. In Colloids and Chemistry**,** the UK groups of R. Ottewill, J.W. White, and in Biology**,** K. Ibel, H. Stuhrmann, Nierhaus, A. Miller, J. Randall, B. Jacrot, P. Timmins. If Maier-Leibnitz’s policy of non-conservative neutron techniques was one major feature of ILL, the mixing of nationalities and novel science initiated by these groups was another.

Rudolph Mossbauer became Director in 1972. When the UK joined ILL Dr Mick Lomer became Associate Director. Lomer, a solid state theorist and former Associate Director of the UK Atomic Energy Research Establishment (AERE) at Harwell, understood the “user” growth of neutron science in the UK. Mossbauer, brought the “user Institut” philosophy to Maier-Leibnitz’s “College” and met the increased demand from France, Germany and the United Kingdom. Lomer must have been helpful- he was a tactful and wise man. A specialist Scientific Council now advised the directors, with distinguished scientists from the three countries. Input from the twelve “Colleges” of ILL scientists, was part of the scientific proposal review. “Air” time at instruments allowed some ILL scientists, to develop science links for their own research (most instrument scientists were on five year contracts). The mixture of different disciplines and nationalities, became a feature at the ILL. Also, at that time, very good proposals from all comers were accepted.

The user-oriented policy changed the *modus operandi* of D11 and other instruments. Mossbauer was clear on this matter. Mick Lomer was already accustomed to user driven science. He had encouraged it at the Harwell reactors as neutron scattering users from UK Universities multiplied. Their enthusiasm came from a belief in the future of the technique but also from a wise attitude that a creative, broad and supportive user base for Harwell was necessary for its own renewal. As the UK community grew, Lomer established sure funding—a Harwell budget line of £50,000 in the late 1960’s. What finally cemented in the user principle in the UK was the Government’s Science Research Council Neutron Beam Research Committee. It was mainly neutron scientists and decided funding and proposal selection. With a slackening ILL budget in 1974, Mossbauer and Lomer created a Technical Department and Project Office under M. Faudou, assisting instrument construction by M. Gobert and Instrument Scientists.

On a three-month sabbatical leave from Oxford in February 1973 I used D11. Making friends was easy at the ILL and was very conducive to collaboration. At that time, Andrew Miller and I were collaborating in Oxford on highly oriented collagen fibres. He was producing samples with quite sharp x-ray meridional reflections from the 664 Å repeat unit in wet collagen (rat tail). We agreed that small angle neutron scattering might be useful for contrast change. I was interested also, in the dynamics of the thin water layers in clays and biology. Tobacco Mosaic Virus (TMV), and the work of Bernal and Fankuchen [8] looked a good target—especially in the oriented tactoid phase above 2 wt%. A very small sample, in buffer with 11 thin tubes, was made and purified for me in Oxford.

**Fig. 3:**
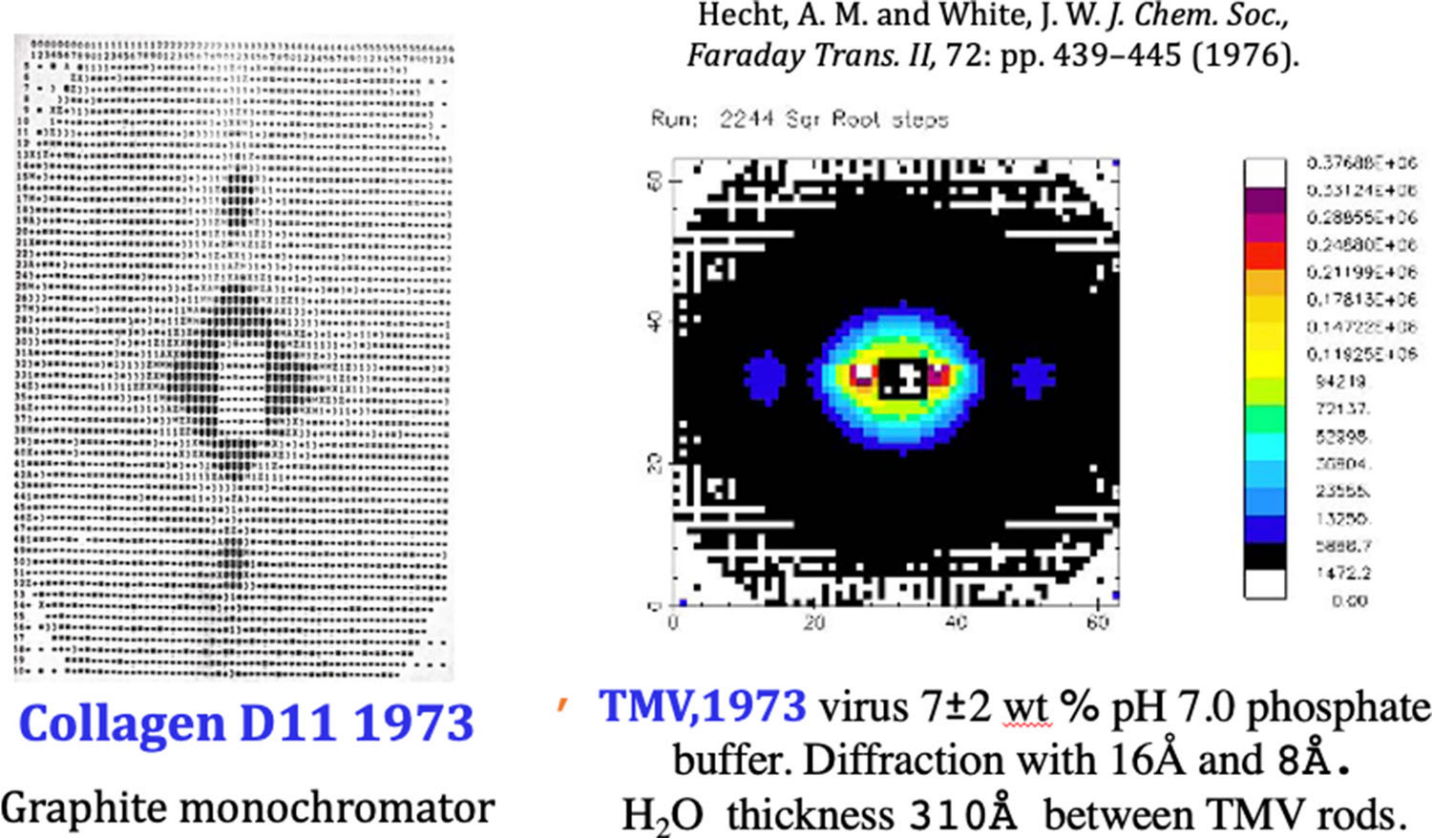
Highly oriented rat tail collagen (D11, 1973) with a graphite monochromator (left) and highly orientated Tobacco Mosaic Virus, 7 ± 2wt% in pH 7.0 PBS, 1973 showing equatorial reflections (right)

Figure 3 is diffraction measured with Konrad Ibel’s and Andrew Miller’s help. The meridional reflection of collagen at 0.01 Å^−1^ is strong and the equatorial reflections from ordered tactoids of TMV in pH7 buffer are clear. The collagen experiment was the first of many by Andrew Miller as Head of the EMBLab outstation at the ILL [7], and the TMV thin inter-virus water was characterised for subsequent quasi-elastic neutron scattering at Harwell [8]. Immediate output was by line printer, a screen photograph or magnetic tape. The results were delightful. In other biological work the value of isotopic contrast variation was demonstrated by Ibel et al. [9–11].

Bernard Jacrot, Associate Director, came down to D11 from building ILL4 to D11 to see these results and recorded our conversation at the time. The following is Ron Ghosh’s translation in “Neutrons for Science” [12].

In the summer of 1973, John White, future director of the ILL, was a scientific visitor, and performed one of the first experiments on a biological sample, collagen and he noted that it diffracted neutrons rather well. We had the following conversation:

B.J.: Yes, collagen diffracts neutrons well; did you make any calculations before the experiment?

J.W: (surprised) Uh, No.

B.J.: It’s always like that with the Anglo-Saxons: they never make preliminary calculations. The Germans, they make such beautiful calculations they have hardly any need to make measurements.

J.W.: And the French?

B.J: We believe it is necessary to do the calculations, but we rarely do it.

John White, who reminded me of this dialogue concluded that research needs to be multinational as at the ILL. I completely agree with this conclusion.

**Fig. 4:**
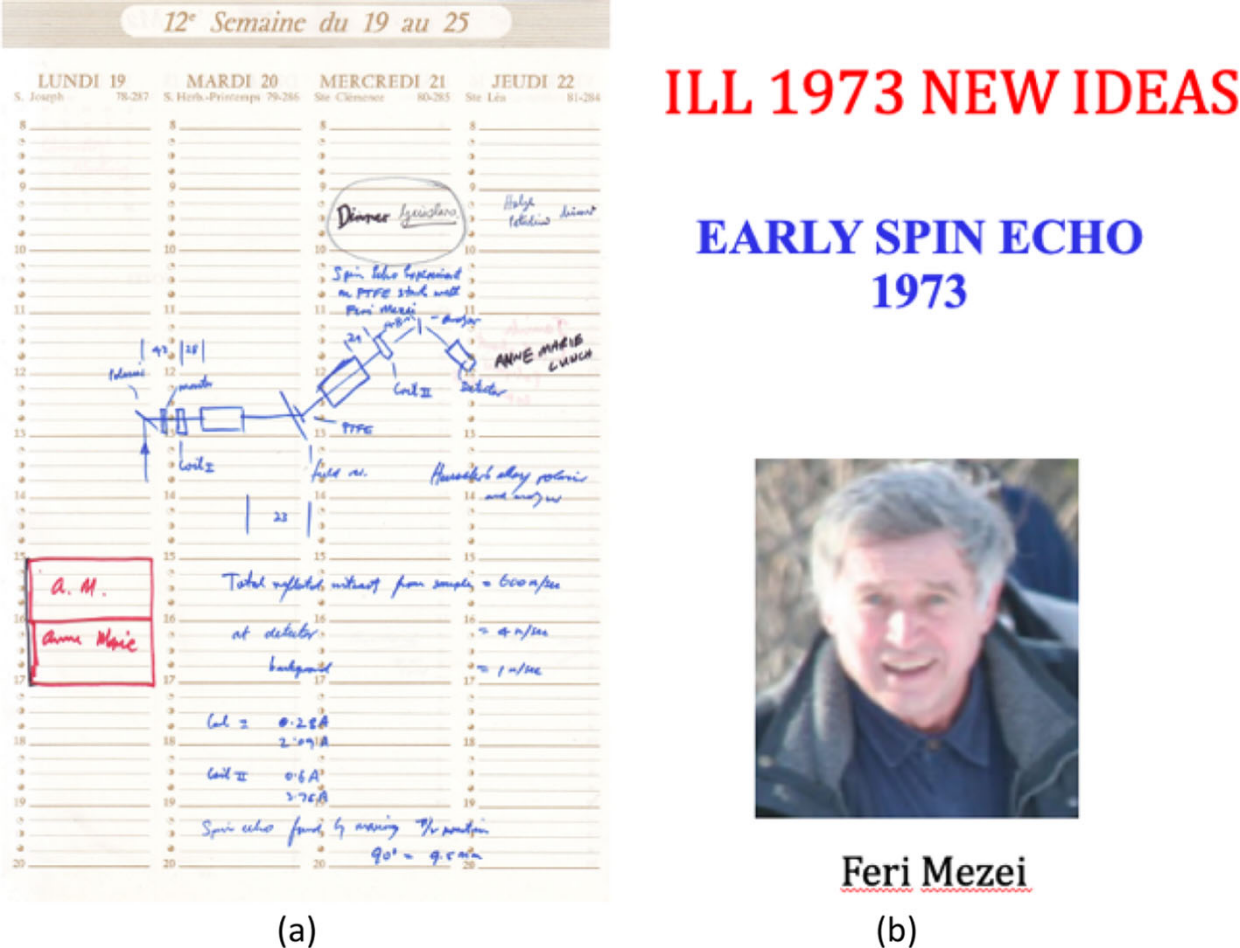
**a** My workbook diagram of Ferenc (Feri) Mezei’s first spin-echo spectrometer at ILL with noted solenoid currents for resonance **b** Feri Mezei

This multinational mixing of ways to do science is another feature of D11. The respect of other scientific cultures is an important role of an international Institut. “Trying out” new ideas and technology was respected. Multi-detectors, monochromators and high capability instruments were being created at ILL. My 3-month sabbatical in February–April 1973, was insightful and productive. Feri Mezei’s first spin-echo spectrometer was built on a table-top in the guide hall and I was glad to join in a small way (Fig. 4).

Feri’s enthusiasm for spin-echo methods was convincing to someone with my nuclear magnetic resonance background. The work was intense. Late one night, after most had left, Feri needed a microcomputer to switch solenoid currents. Electronics was freely available and he wired-up a PDP 11 computer there and then to run the device. I had no hesitation to recommend John Hayter, one of my best post docs ever, to the Directors, when a post came up, to help Feri build IN13—the first operating spin-echo spectrometer. Regrettably, my experiment with Feri on the table-top did not work! The 8 °C phase transition in Teflon—the sample being cooled with nitrogen boil-off in a plastic bag–did not produce any fluctuations that could be measured.

## Difficult decisions

With British entry, rising operational costs and an abundance of new ideas the French, German and British partners asked for reviews of the expected budgetary needs to maintain the obvious growing success of the Institut. An estimate of the expected reactor lifetime was also requested. These matters were the origin of the “Second Wind” “Deuxième Souffle”. Preparation occupied the years 1977 to 1979. The “Long-Term Prospects for the lnstitut and Renewal Programme” study required estimates of the needs for re-investment in three areas: the instruments, the reactor, guides and infrastructure. The consequences of low investment in new instruments over the previous few years and mounting running costs were presented. Areas of weakness in the instrument sector were appraised and three documents formed the basis of the draft report on future prospects were submitted to the ILL Steering Committee in May 1977. This submission resulted in the ILL being asked to further define and cost key components of a renewal programme, a “second souffle”. In the summer of 1977, working groups were active, defining new instruments, improved sources, and new neutron technology.

**Fig. 5:**
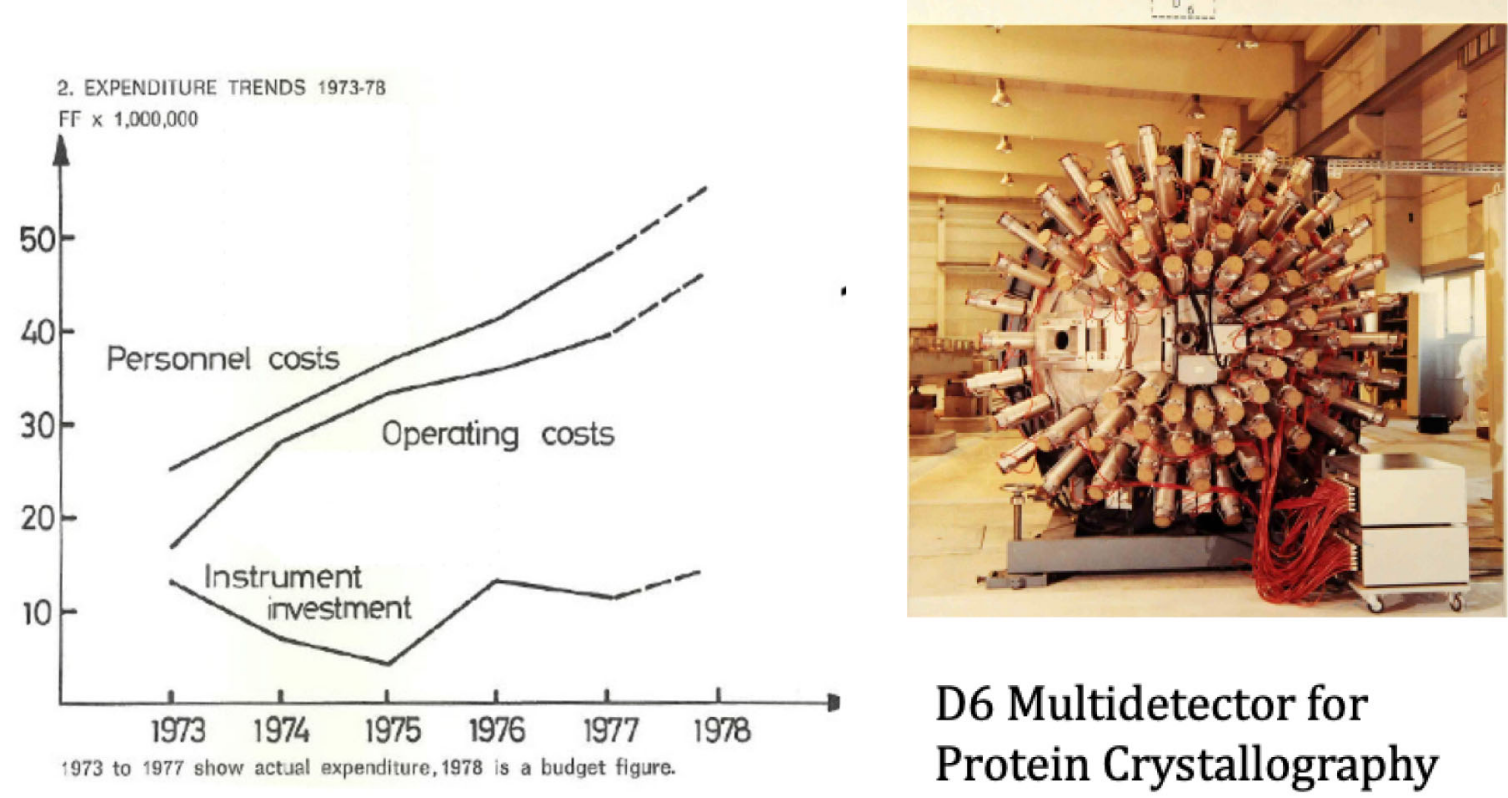
Rising, operating and personnel costs, and the D6 Multi-detector for protein crystallography- closed with three other instruments

The best instrument proposals were studied, the operation of current instruments examined, the priorities and development needs in the reactor, the neutron sources, and the maximisation of beam intensity and detection to maintain leadership in the field. There was a mixture of grief and excitement in this process—grief in closing some instruments, the avoidance of sharing beam tubes—to maximise flux for some, the increased sharing of guides. Closing instruments which had been lovingly built and adjusted but for which, the hopes of performance were beyond the current technology was hard, for example D6 illustrated in Fig. 5b. The most sensitive part of the radial detectors was difficult to locate on the crystalline Bragg spots. Mixed with these disappointments was excitement for those working with new monochromators, supermirrors, multi-detector improvements—all of which had to be paid for.

A final document “Report on Long Term Prospects for the Institut Max von Laue—Paul Langevin, Grenoble, France” was submitted to the Steering Committee on 2 December 1977. The programme was oriented towards the “best”, with three underlying principles (a) No compromise of beam intensity at instruments (b) Complementarity with national sources and (c) Computing power at instruments and central to all of this was the rising cost of operation, while the availability of new technology was also in mind.

In 1978–79 there was much to and from consultation with the national partners about the size and timing of the budgetary injection. For example, the need for a fast or protracted injection. To keep the creative momentum the Director advocated a fast process. This, discussed at a meeting in Paris with CNRS and CEA representatives, was accepted after compromises on both sides and patience. It was an exhausting but energising time. The process was heartened by a number of features, for example, the goodwill of our German partners. About then the Head of Administration at ILL was offered a good job in Germany. In response to our advertisement, the Bundesministerium für Forschung und Technologie (BMFT) offered two candidates—favouring one. After the interview at the ILL, we favoured the other. So, that Friday, the ILL Director contacted the BMFT to say he would come to Bonn to explain. He was received with great courtesy. The subject of the choices was not raised. All the questions and discussion were about the Deuxième Souffle!

**Fig. 6:**
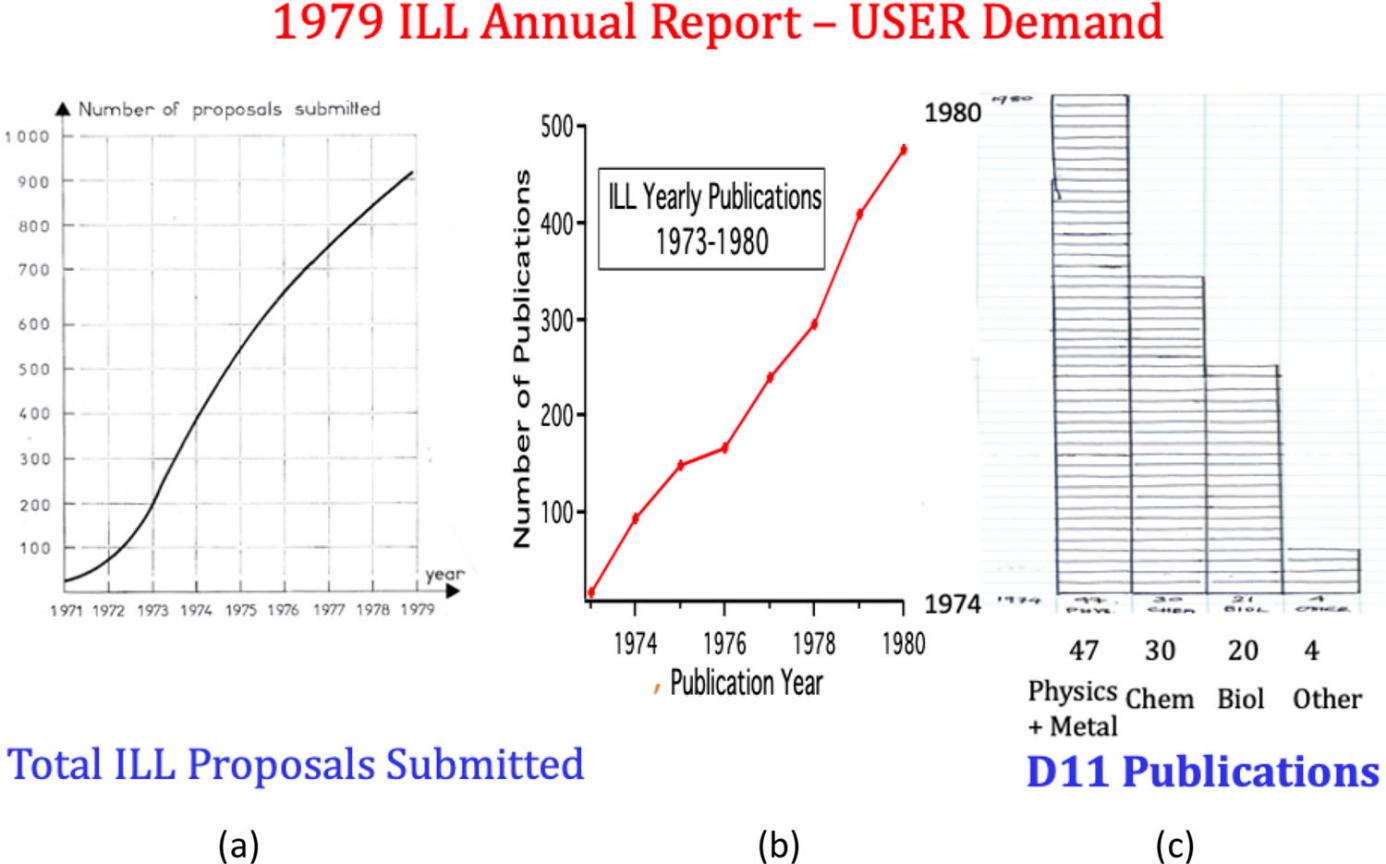
**a** Continuous increase in demand **b** Annual publication rates 1974–1980 and **c** Diversity of subjects interested in using D11

The Institut was performing well in 1979—as judged by number of proposals, the number of publications and the growing diversity of their subjects, (Fig. 6). In Fig. 6c, D11’s spread of interest had continued. Metal physics—including superconductivity was the dominant subject, now closely followed by chemistry and biology. This spread continues—we show below how these interests grew further over the 10 years after the deuxième souffle.

## A golden period

There have been a number of “golden periods” at the ILL. That between 1973 and the early 1980s was one for D11 and the whole Institut. User orientation and the actions towards getting the “Second Souffle” were “digested”. Funding was found for new inventions from a tight budget. Supermirrors became available, an engineered spin-echo was started, IN7 moved from concept to design. The instruments for diffraction and inelastic scattering—enhanced by the detector and monochromator programs—flourished—all to the benefit of new users. Newly conceived programs such as strain testing and neutron contrast imaging came into view.

Not to be forgotten were the advances in fundamental physics such as accurate determination of the neutron lifetime, the lower limits of the neutron dipole moment and the spinor nature of neutrons. Thankfully, the theory division was strong—as much for the benefit of the solid state and the soft matter science communities under a succession of distinguished leaders.

The informal way in which creativity worked is worth noting. The coffee room on the fifth floor of ILL 4, with its blackboards, was a twice-daily hub—governed by Carmen at the coffee machine. In the spirit of Maier-Leibnitz’s idea, parties or celebratory “pots” were good places to discuss. I remember the night that the idea for IN7 had arisen around the barbecue fire at Til von Egedi’s house in 1977. The concept came with our discussion about the way that a rotating mosaic monochromator crystal “scoops up the phase space” and fires it in a particular direction. Scherm and Carlile (future directors of ILL), White, Suck and others were part of that conversation.

The final agreement to fund the Deuxièms Souffle was made at the 1979 May Steering Committee. The programme allowed bursts of activity for projects past 1980 and neutron and reactor technology that had been initiated between 1977 and 1980. Technical and scientific staff were recruited, a new cold source provided and new buildings for computing and the User community.

**Fig. 7:**
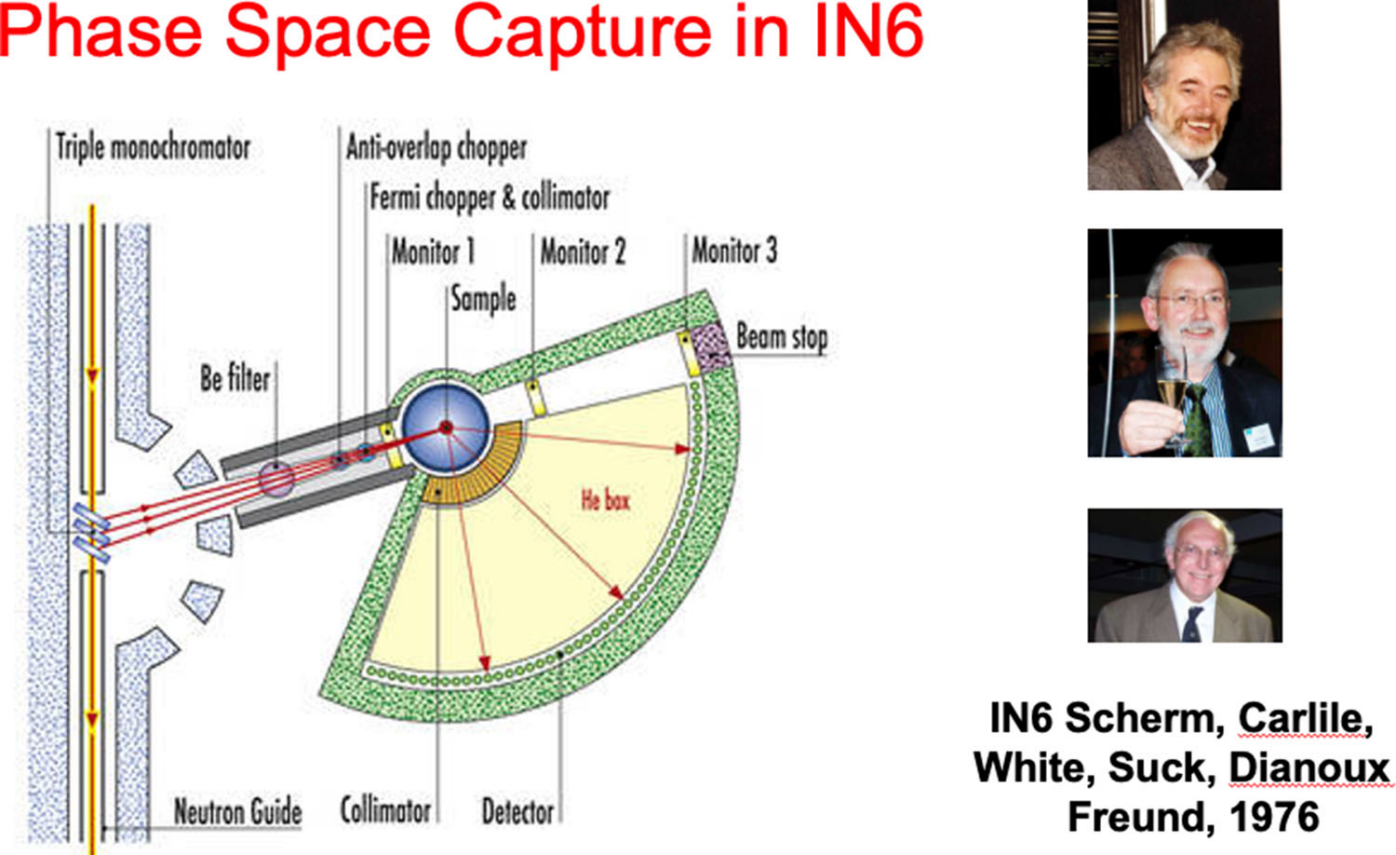
IN7 (later to become IN6) triple use of the neutron guide beam, focusing and time of flight analysis as conceived in 1977

**Fig. 8:**
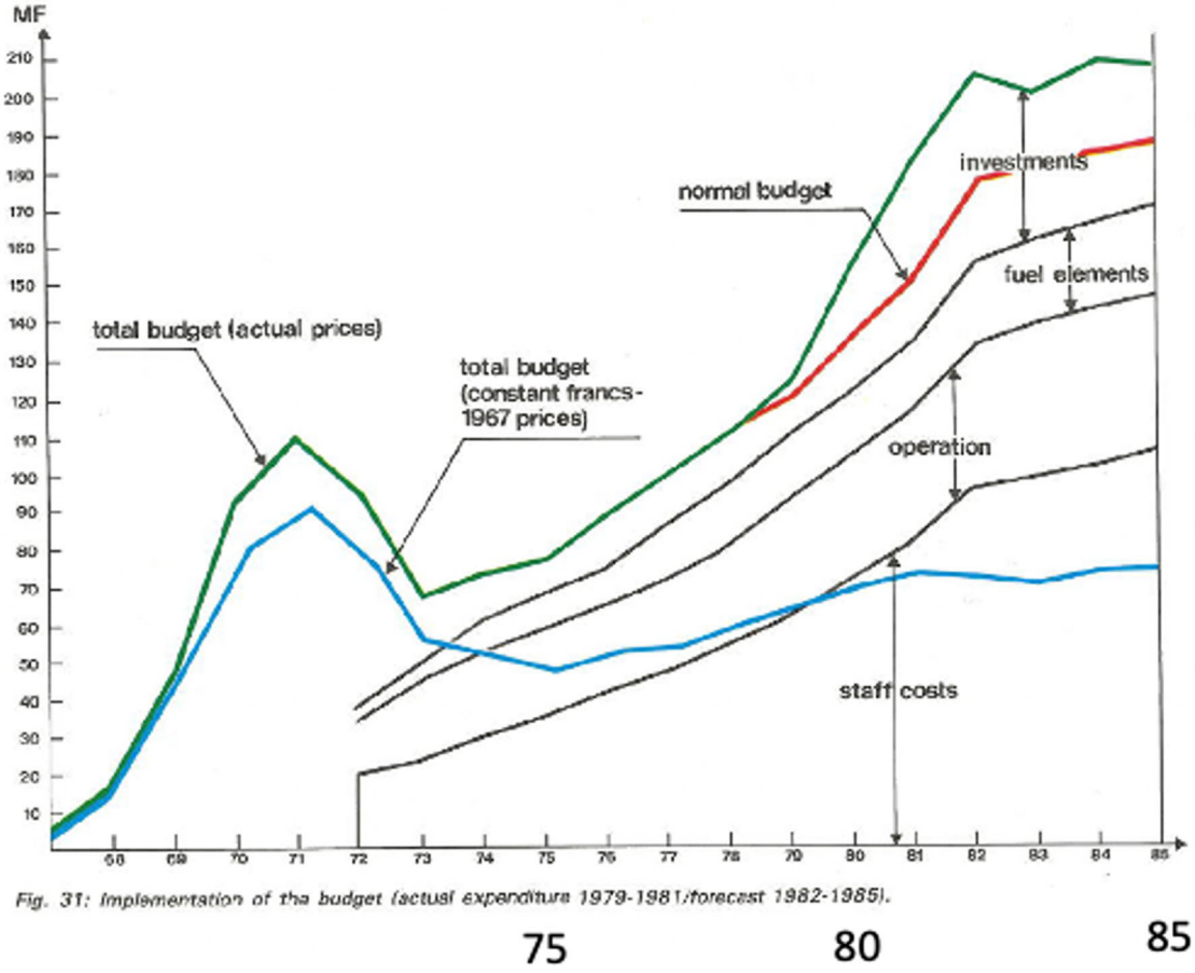
The ILL budget to 1985 showing the continuing supplement from the “Deuxieme Souffle” above the red line. The blue line shows the budget in 1967 francs

**Fig. 9:**
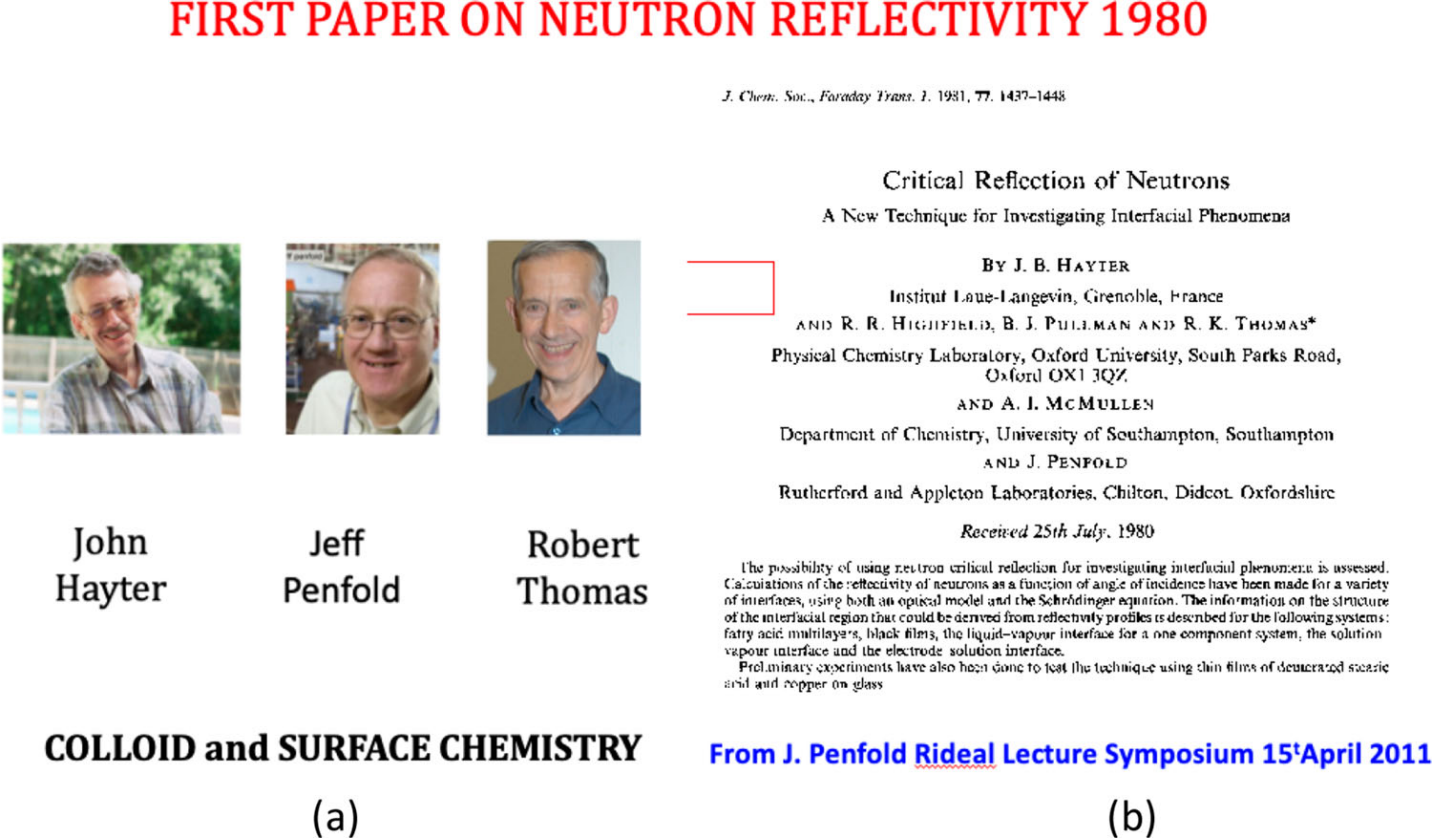
**a** J.B. Hayter, R. K. Thomas and J. Penfold and **b** Neutron reflectivity-first paper from ILL 25 July 1980 [13]

Figures 7, 8 and 9 illustrate the impact of new science from methods conceived in the “Deuxième Souffle preparation. The IN7 wide Q range, coupled with high energy resolution and intense beams made it one of the most highly used at ILL. It has made neutron inelastic scattering feasible for chemists, physicists and some biologists. Figure 9 recognises the extensive new field of interfacial physical chemistry, surfactant science, polymers and biology coming from fundamental studies at ILL of neutron reflectivity—together with what can be done in the bulk at D11—both with isotopic contrast variation.

## Australia

After 1985, in the Australian National University (ANU) the growing chemical usefulness of D11 and the lack of any equivalent in Australia, was a motive to build a SAXS instrument that would be at least as good. Offered an Argonne Fellowship by the University of Chicago and Argonne National Laboratory at the same time, the SANS instrument at Argonne created by Jack Carpenter was available. Lennox Iton collaborated on the use of neutron contrast variation to study zeolite nucleation by template molecules with SANS.

The ANU not only allowed acceptance of the Fellowship but also provided about $1 million for constructing an excellent SAXS instrument. The wisdom of Uli Arndt at Cambridge and Gabriel at the ILL was indispensable. An excellent technician, Trevor Dowling at the Research School of Chemistry was in charge. We were fortunate that Thomas Zemb was in Canberra also—he impressed upon us the value of the absolute scale of intensity. The SAXS instrument eventually had a 2 m flight path sample to detector, Huxley Homes optics, all phase space matched to the”big-wheel” rotating anode generator, GX 13. The performance, pre-synchrotron, was one of the best in the world in resolution and intensity, with many university users and the first National Small Angle Conference held at ANU in 1989.

The possibility of building a SANS machine at the Lucas Heights HIFAR reactor became a reality. The then Director of ANSTO Dr David Cook became quite interested in a lecture I gave in 1987 to the ANZAAS (Australian and New Zealand Association for the Advancement of Science). The lecture explained isotopic contrast variation in SANS and the triangulation experiments on ribosome in ILL and the USA. AUSANS-the instrument, was completed in 1992, somewhat delayed because ANSTO decided that it should build (rather than buy) the 2-dimensional detector—against my advice.

Peter Timmins, in his lecture, traced the continuous upgrading of D11 mostly under the supervision of Peter Lindner and Roland May. From 1979 (when the first new detector was installed) to 2012, where the scope of this paper ends, the ease of the instrument’s use and its momentum transfer range, were continuously improved. With a new detector, tank project and guide replacement the diversity of subjects and accuracy on the absolute scale were achieved.

## A Legal Dispute

Nothing but the best was needed to resolve a legal dispute between two companies in Australia in 2003. D11 provided this. It was to be the first experiment at ILL after 20 years of absence of my group. After ten years work on ammonium nitrate explosives using small angle x-ray scattering at our GX-13 Huxley Holmes 2 m camera, aided by SANS work in USA and the UK, we had become quite familiar with emulsion chemistry. I was approached to become an expert witness in the Federal Court of Australia.

The case to be tried was whether a sheep drench—Q-Drench—infringed a patent held by a large, Australia-wide agricultural products company. The defendant was a small local firm. The matter finally hung on whether the defendant’s product was an emulsion (as specified in the patent application) or microemulsion or micellar solution. A definitive proof was required and documentation precise.

The court case required much experimentation for Affidavits for both sides—the plaintiff conducting many tests, including high resolution NMR and ultra-centrifugation on Australia’s most powerful centrifuge. The clear liquid did not “break” which showed that the surfactant mixture of Tween and benzoyl alcohol was likely to be a microemulsion phase. For the plaintive we decided that a test on D11, the undoubted best SANS instrument in the world, would solve the problem. ILL’s Industrial applications programme allowed this.

**Fig. 10:**
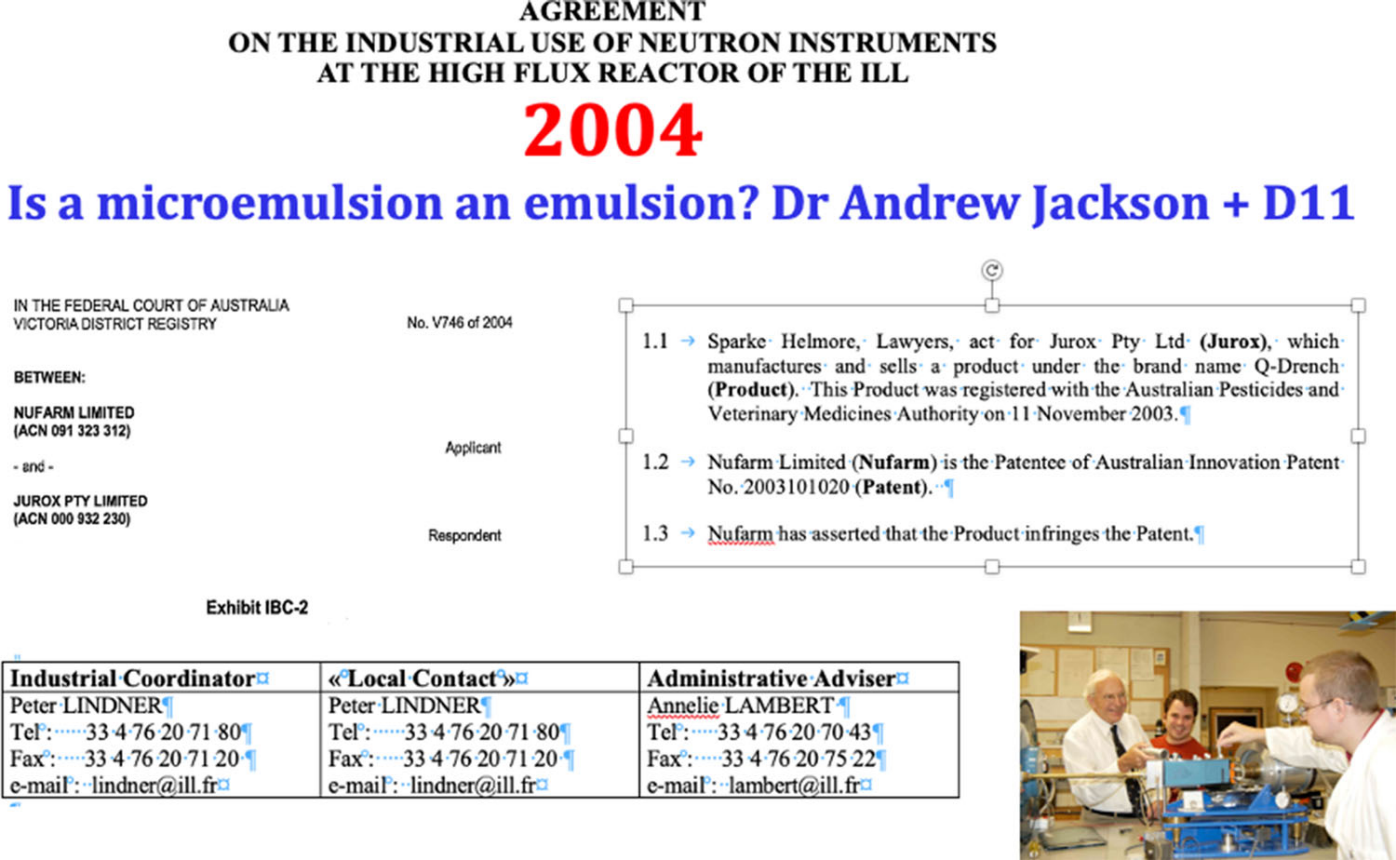
Agreement for a confidential “industrial” project with the ILL. Photo shows Andrew Jackson adjusting the focus on the GX-13 SAXS instrument at ANU

**Fig. 11:**
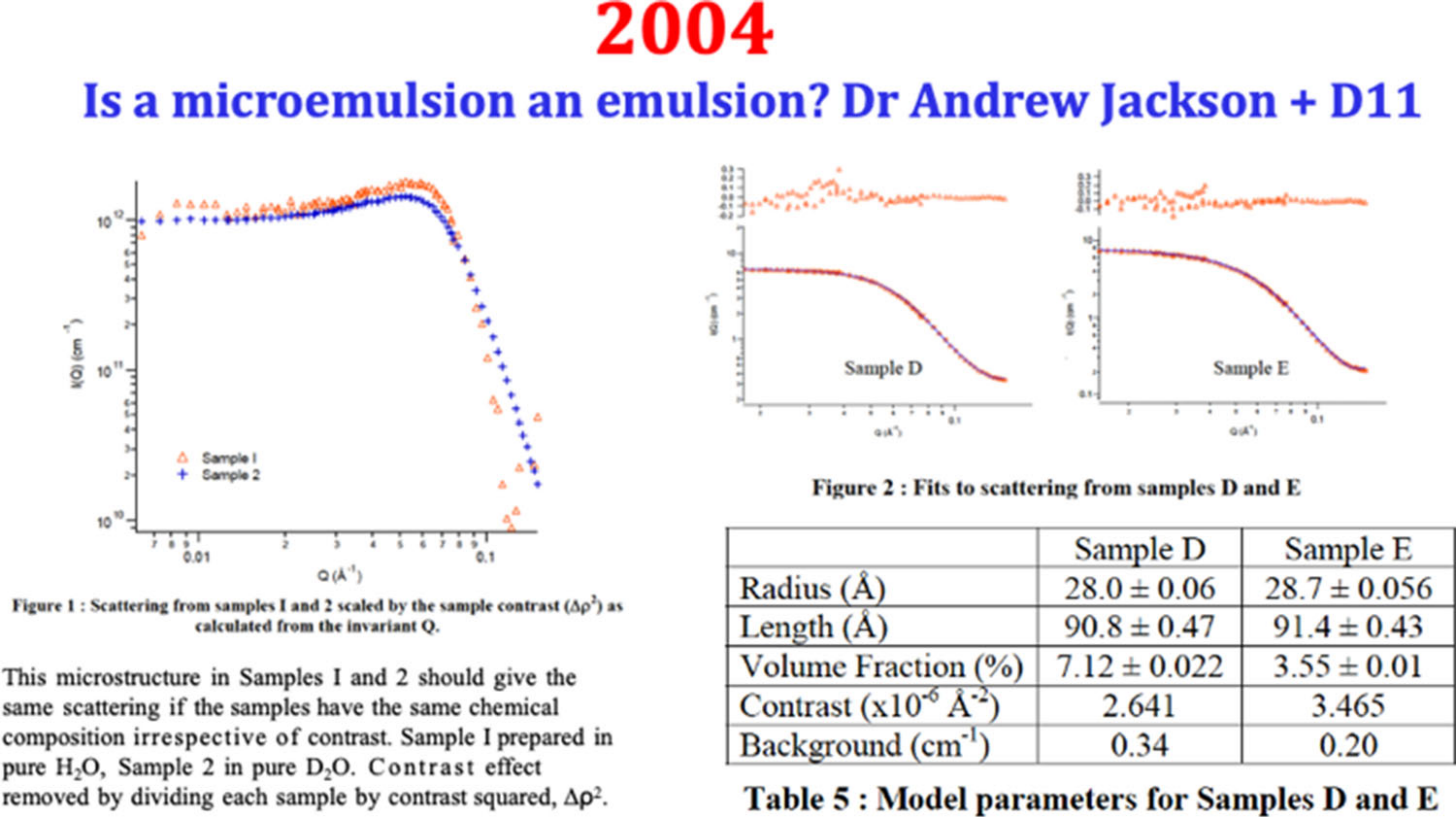
Andrew Jackson’s data-fitting of the SANS from Q-Drench micellar phase

Dr Andrew Jackson was working with us in Australia. His skill to make a thorough experiment on D11 was trustworthy to find the size and structure of this microemulsion. Five samples were run on D11 with the help of Peter Lindner and the D11 team at that time (Figs. 10, 11). A small micellar phase was quantitatively characterised and the case was won by the defendant small company. The win should be attributed to D11 and the team and Andrew—a lot hung on this judgement. The utter clarity required for the affidavits and the forensic nature of the legal cross-examinations was salutary.

**Fig. 12:**
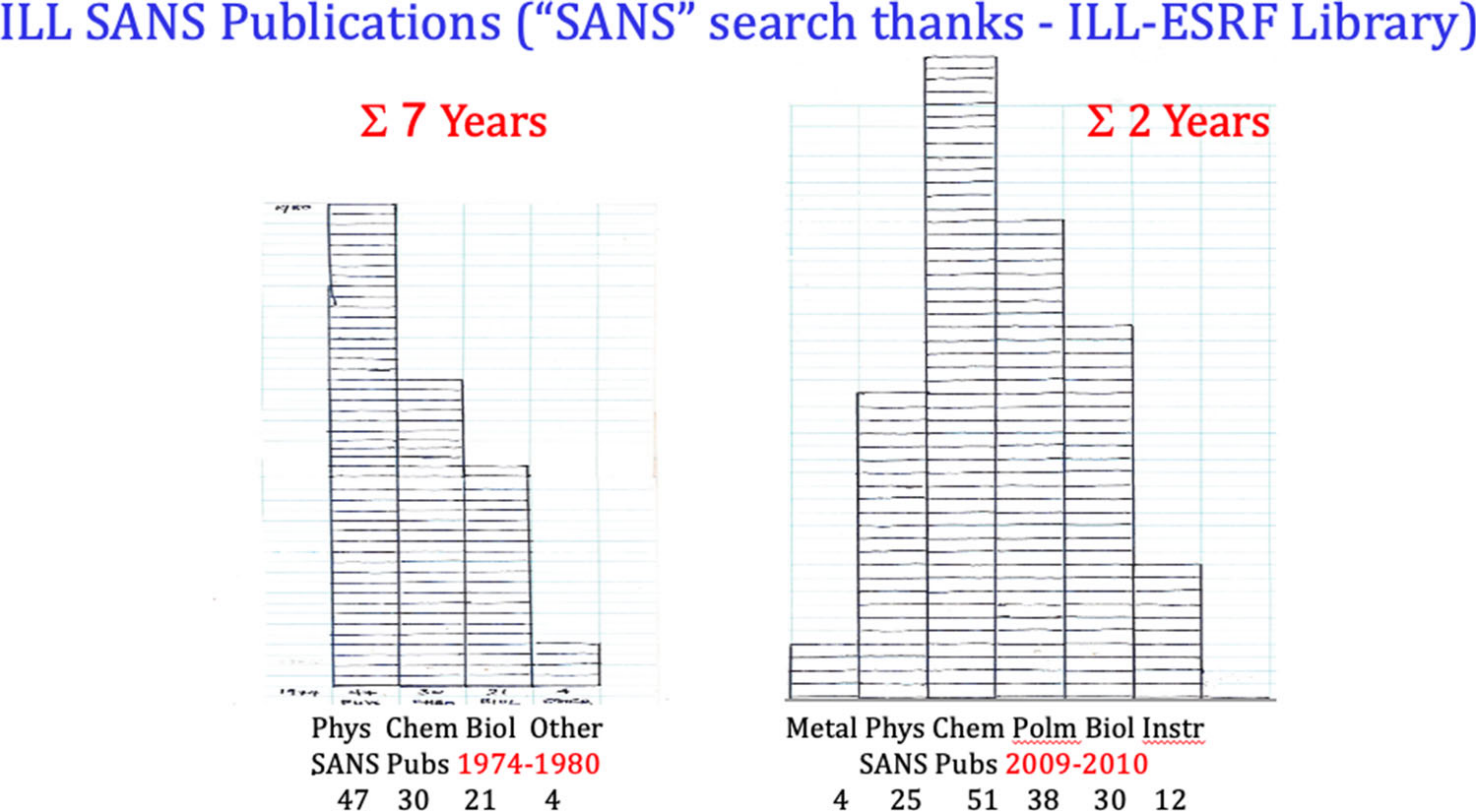
Comparison, from “ILL SANS Publications” (ILL-ESRF Library) [14] of the breadth of science done by small angle scattering at ILL in 1970, and in 2009–2010 Annual reports

Thoughtful improvements in instrumentation always introduce new science. This continues at D11 and is the record of science in general. This stimulation is accompanied by the reverse—that newly conceived science, (by theory or experiment), itself, sometimes requires new instrumentation in order to probe further. Figure 12 graphically illustrates, by publications, some achievements for D11 after the 2008–2010 D11 renewal.

**Fig. 13:**
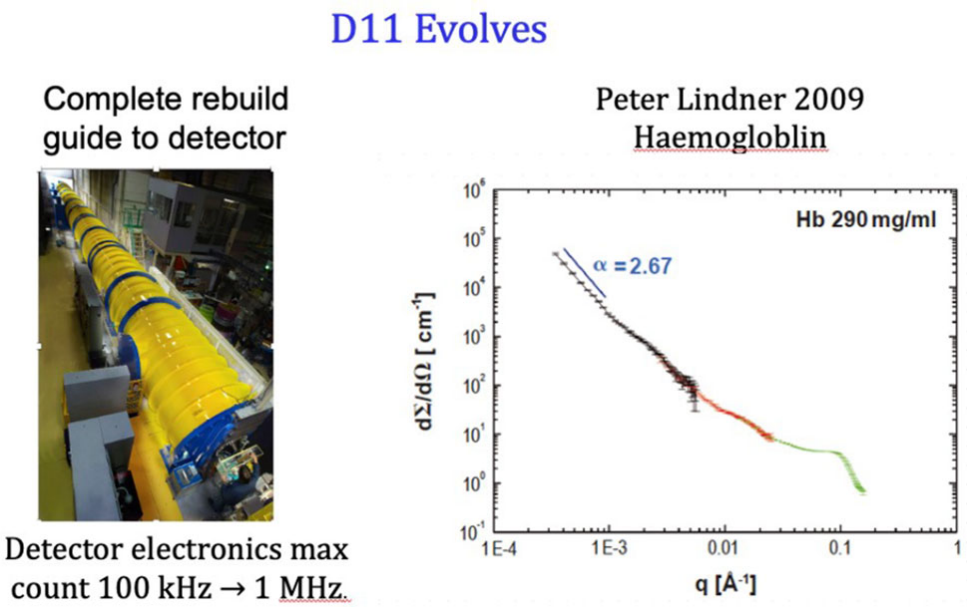
D11 improvements in the 2008–2009 refurbishment: (left) the new vacuum tank with computer-controlled detector movement and (right) extended *Q* range of data and accuracy of data merging

What will happen next is for the next “chapter”? The ground for this was well prepared by the refurbishments of 2009–2010 (Fig. 13) and the Institut’s willingness to fund.

At the time of writing new changes are projected for the instrument. Best wishes for the future to D11 and its team and a Happy 50th Birthday. In conclusion I thank my many former colleagues, particularly those at D11 and ILL for their innovative science and their courage to “try new things”. John White was the Director of ILL 1977–1980.
